# Study Protocol- Lumbar Epidural Steroid Injections for Spinal Stenosis (LESS): a double-blind randomized controlled trial of epidural steroid injections for lumbar spinal stenosis among older adults

**DOI:** 10.1186/1471-2474-13-48

**Published:** 2012-03-29

**Authors:** Janna L Friedly, Brian W Bresnahan, Bryan Comstock, Judith A Turner, Richard A Deyo, Sean D Sullivan, Patrick Heagerty, Zoya Bauer, Srdjan S Nedeljkovic, Andrew L Avins, David Nerenz, Jeffrey G Jarvik

**Affiliations:** 1 Comparative Effectiveness, Cost and Outcomes Research Center, University of Washington, Seattle, WA, USA; 2 Departments of Family Medicine and Internal Medicine, Oregon Health Sciences University, Portland, OR, USA; 3 Department of Anesthesiology, Perioperative and Pain Medicine, Brigham and Women's Hospital, Boston, MA, USA; 4 Division of Research, Kaiser Permanente Northern California, Oakland, CA, USA; 5 Neuroscience Institute, Henry Ford Hospital, Detroit, MI, USA

## Abstract

**Background:**

Lumbar spinal stenosis is one of the most common causes of low back pain among older adults and can cause significant disability. Despite its prevalence, treatment of spinal stenosis symptoms remains controversial. Epidural steroid injections are used with increasing frequency as a less invasive, potentially safer, and more cost-effective treatment than surgery. However, there is a lack of data to judge the effectiveness and safety of epidural steroid injections for spinal stenosis. We describe our prospective, double-blind, randomized controlled trial that tests the hypothesis that epidural injections with steroids plus local anesthetic are more effective than epidural injections of local anesthetic alone in improving pain and function among older adults with lumbar spinal stenosis.

**Methods:**

We will recruit up to 400 patients with lumbar central canal spinal stenosis from at least 9 clinical sites over 2 years. Patients with spinal instability who require surgical fusion, a history of prior lumbar surgery, or prior epidural steroid injection within the past 6 months are excluded. Participants are randomly assigned to receive either ESI with local anesthetic or the control intervention (epidural injections with local anesthetic alone). Subjects receive up to 2 injections prior to the primary endpoint at 6 weeks, at which time they may choose to crossover to the other intervention.

Participants complete validated, standardized measures of pain, functional disability, and health-related quality of life at baseline and at 3 weeks, 6 weeks, and 3, 6, and 12 months after randomization. The primary outcomes are Roland-Morris Disability Questionnaire and a numerical rating scale measure of pain intensity at 6 weeks. In order to better understand their safety, we also measure cortisol, HbA1c, fasting blood glucose, weight, and blood pressure at baseline, and at 3 and 6 weeks post-injection. We also obtain data on resource utilization and costs to assess cost-effectiveness of epidural steroid injection.

**Discussion:**

This study is the first multi-center, double-blind RCT to evaluate the effectiveness of epidural steroid injections in improving pain and function among older adults with lumbar spinal stenosis. The study will also yield data on the safety and cost-effectiveness of this procedure for older adults.

**Trial Registration:**

Clinicaltrials.gov NCT01238536

## Background

Lumbar spinal stenosis is one of the most common causes of low back pain among older adults and can result insignificant disability [1]. The cause of spinal stenosis is often multifactorial and the clinical presentation can be variable. Degenerative changes in the spine such as spondylosis, facet arthropathy, disc degeneration, and scoliosis may all contribute to the development of spinal stenosis. The symptoms of lumbar spinal stenosis range from low back pain to neurogenic claudication with lower extremity pain, weakness or sensory changes and are often aggravated by walking. Because spinal stenosis can affect the central canal as well as the lateral recesses and intervertebral foramen variably, symptoms can involve single or multiple myotomes and dermatomes. Because the causes of spinal stenosis are most frequently degenerative changes, the symptoms often, but not always, worsen over time. Lumbar spinal stenosis is often associated with poor patient health outcomes and functioning, high resource utilization, and substantial payer, patient, and health-system costs.

Despite the prevalence of symptomatic lumbar spinal stenosis among older adults, treatment remains controversial, with limited comparative effectiveness evidence. Common treatments include conservative measures such as non-steroidal anti-inflammatory drugs (NSAIDS), activity modification, and physical therapy, as well as more invasive treatments such as epidural steroid injections (ESI) and surgery [1]. Although surgery provides some benefit to certain individuals with spinal stenosis [2,3], the results of surgical treatment tend to be modest and may be accompanied by short and long term postoperative surgical complications [4]. Consistent with the general trend of using more minimally- invasive treatment approaches in health care, ESIs are being used with increasing frequency as a less invasive, potentially safer and more cost-effective treatment. However, there is a lack of evidence examining the efficacy, safety, and effectiveness of ESI for symptoms of spinal stenosis. Despite the lack of data demonstrating effectiveness in patients with spinal stenosis, an estimated 25% of all ESIs performed in the Medicare population and 74% of ESIs in the Veterans Affairs system are for spinal stenosis [5,6].

A few cohort studies suggest that ESI may provide short-term pain relief [7,8]. However, the results of RCTs have been less favorable. One early RCT [9] demonstrated no advantage of ESI over saline or local anesthetic injections. However, some argue that the poor results of this trial were a consequence of inadequate technique and do not reflect the current practice of performing ESI with fluoroscopic guidance to ensure appropriate placement of the steroid medication [10].

Only one RCT of fluoroscopically-guided ESI compared to injections with local anesthetic alone has been published [11]. This study, while showing improvement in each group, found no advantage of the steroid injection over an injection of local anesthetic alone [11]. However, this study suffered significant methodological limitations including lack of statistical power, no primary outcome measure, un-blinding of patients and researchers and a high dropout rate (21/60 patients).

The overall objective of this multi-center, double-blind RCT is to evaluate the effectiveness of ESI plus local anesthetic versus local anesthetic alone in improving pain and function among older adults with back pain and lumbar spinal stenosis. We hypothesize that patients randomized to receive ESI with local anesthetic will show significantly greater improvement in pain and function compared to patients randomized to receive local anesthetic only. We will also examine whether ESI is differentially effective in improving pain and function for different patient subgroups (African-Americans vs. Caucasians and patients undergoing a transforaminal vs. interlaminar approach). Finally, we will compare resource use and costs in the two treatment groups as well as incremental cost-effectiveness and incremental cost utility from both payer and societal perspectives.

## Methods/design

### Study design

The Lumbar Epidural steroid injections for Spinal Stenosis (LESS) study is a double-blind RCT of ESI plus local anesthetic injections versus local anesthetic injections for the treatment of pain associated with lumbar spinal stenosis in older adults. Patients, study investigators, and the research staff collecting data will remain blinded to patient treatment allocation.

**Figure 1:**
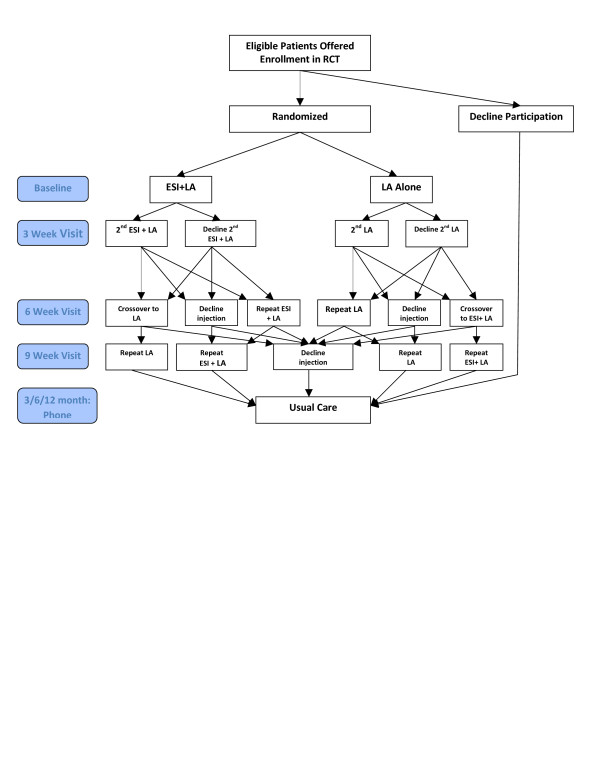
**Study Flow Chart**.

The patient follow-up has three distinct phases--from randomization and baseline injection to the primary endpoint at six weeks, from optional crossover at six weeks until nine weeks, and the follow-up period from nine weeks to one year after randomization (see Figure 1). Randomization will occur just prior to the first injection. There is a follow-up visit at three weeks from randomization, at which time the participants may have a second injection if they choose, and blood is drawn to obtain cortisol, HbA1c and fasting glucose levels.

As an inducement to study enrollment, we will allow crossover after the primary outcome data have been collected at six weeks. Participants may crossover to the other blinded treatment arm if they believe they had insufficient benefit from the first two injections. Also at six weeks, patients will return to discuss potential crossover and to again have blood drawn for cortisol, HbA1c and fasting glucose measures. Those who choose not to crossover at six weeks will be allowed to have one additional injection of the same type as originally assigned at the nine-week time point, if they desire. After nine weeks, patients and physicians will be allowed to make treatment decisions per usual care. This may include ESIs or other treatments as determined by the patient and physician.

### Subjects and setting

The LESS trial is part of a research program to study older adults with back pain, the Back pain Outcomes using Longitudinal Data (BOLD) project. The project involves building a registry designed to capture data from patients enrolled in three large integrated health care systems in the United States. The BOLD registry will provide a mechanism for collecting outcomes data on a large number of patients with back pain and will serve as a portal for entry into studies such as the LESS trial. We will also be recruiting patients from several academic medical centers. Each participating clinical site obtained local Institutional Review Board (IRB) approval prior to subject recruitment. Patients with spinal stenosis referred for an ESI are asked if they would like to participate in the LESS trial. Prior to recruitment, the treating physician determines whether the patient is an appropriate candidate for ESI based on history, physical examination and imaging studies. The physician introduces the study at the end of this consultation if the patient is deemed an appropriate candidate for ESI. Each potential participant is invited to view an educational video that describes the study and provides detailed information about the procedures and implications of participation in the study. Patients provide written informed consent when enrolling in the study.

### Eligibility criteria

Study inclusion and exclusion criteria are listed in Table 1. Inclusion criteria were chosen to include older adults with at least moderate pain and disability related to neurogenic claudication from central spinal stenosis. Subjects will need to have CT or MRI evidence of central lumbar spinal stenosis (mild, moderate or severe) according to the criteria of Boden et al. [12] Patients with lateral recess or foraminal stenosis without the presence of central canal stenosis are not included in this study. The exclusion criteria were chosen for safety concerns as well as to eliminate known confounders that might influence the results of the study or hinder participation and follow-through with study protocol. In addition, we are excluding patients with a history of lumbar fusion or decompression surgery (i.e. laminectomy) as well as interspinous process spacer procedures as this may impact the injection technique as well as outcomes. To ensure that treatment results are not influenced by prior ESI use, we are also excluding patients with a history of lumbar ESI within the last six months.

**Table 1:** Inclusion and Exclusion Criteria

Inclusion Criteria	Exclusion Criteria
Pain in the low back, buttock, and/or lower extremity (pain NRS > 4) with standing, walking and/or spinal extension (buttock/leg > back pain)	Cognitive impairment that renders the patient unable to give informed consent or provide accurate data.
Roland-Morris score of at least 7	Fibromyalgia diagnosis, chronic widespread pain, lower extremity amputation, Parkinson's, head injury, stroke, other neurologic conditions.
Mild-moderate-severe lumbar central canal spinal stenosis identified by MRI or CT according to the criteria of Boden et al. [12]	Severe vascular, pulmonary or coronary artery disease that limits ambulation including recent myocardial infarction (within the last 6 months).
Lower extremity symptoms consistent with neurogenic claudication	Spinal instability requiring surgery.
Must be able to read English and complete the assessment instruments	Severe osteoporosis as defined by multiple compression fractures or a fracture at the same level as the stenosis.
Age 50 or older	Metastatic cancer.
	Excessive alcohol consumption or evidence of non-prescribed or illegal drug use as determined by the TICS screening questionnaire (1 or more positive answer).
	Possible pregnancy or other condition that precludes the use of fluoroscopy.
	Concordant pain with internal rotation of the hip (or known hip joint pathology).
	Active local or systemic infection.
	Allergy to local anesthetic, steroid or contrast.

### Baseline and follow up assessments

Patients with spinal stenosis who are being referred for an ESI as part of their treatment plan are invited to participate in this study by the treating physician. Interested patients are then screened for eligibility by the research coordinator at the site. If eligible, the potential participant is asked to participate in the study and to complete the informed consent process. A study physician reviews each potential participant's imaging studies to determine the eligibility criterion of presence of central spinal canal stenosis and to categorize the stenosis as mild, moderate or severe.

Baseline questionnaires assess demographic, clinical, and disease-history information and include patient self-report measures of pain, function, fear avoidance [13], and pain catastrophizing [14]. We also ask patients at baseline about their preferences for assignment to the ESI versus the control intervention, and to rate their expectations of pain relief. All medications used for pain at baseline are documented by medical record review as well as co-morbidities measured by the Charlson Comorbidity index [15].

Our primary outcomes are measures of function and pain. These include the Roland-Morris Disability Questionnaire, a back pain-specific functional status questionnaire adapted from the generic Sickness Impact Profile (SIP). It consists of 24 yes/no items, which represent common dysfunctions in daily activities experienced by patients with low back pain. The other primary outcome measure is pain recorded on an 11-point numerical rating scale(NRS) measuring average leg and back pain (separately) in the past week. The primary outcome measures are completed at 3 days, 14 days, 3 weeks, 6 weeks, and 3, 6 and 12 months post-randomization.

Secondary outcome measures include the Brief Pain Inventory (BPI) Interference scale [16], the Patient Health Questionnaire (PHQ-8)measure of depression [17], Generalized Anxiety Disorder (GAD-7) measure [18], Swiss Spinal Stenosis Questionnaire (SSSQ) [19-21], and the EQ-5D [22]. These are administered at all follow-up time points with the exception that the SSSQ, BPI, PHQ-8, GAD-7 and EQ-5D are not administered at Days 3 and 14.

At baseline and at three weeks and six weeks post-injection, we draw blood to measure HbA1c and fasting blood glucose and morning blood cortisol levels to monitor for hyperglycemia and adrenal suppression following steroid administration. Because patients are blinded to treatment assignment, these are checked in all patients regardless of treatment received. In addition, at each in-person visit, we check blood pressure and weight because these can also be affected by steroid administration.

Other measures include patient-completed resource utilization questionnaires to collect information on prescription and over-the-counter medication use, medical visits outside the health plan, and other patient burdens and costs during four 3-week periods throughout the 12-month study. Resource use and costs for utilization within each health system are available through the electronic health information systems (pharmacy and medical use).

### Randomization

Treatment assignments are stored centrally in a secure database at the study data coordinating center in Seattle, Washington. For each recruitment site, we utilize permuted-block randomization to achieve roughly balanced groups. We use random blocks of size 4, 6, 8, or 10 that are not divulged to the research assistants involved in recruitment in order to avoid bias in the recruitment process. Two pre-filled opaque syringes are obtained for every study procedure, one filled with the steroid and the other with the local anesthetic. The randomized treatment assignment is obtained via a password-protected study website by the clinical nurse or assistant not involved with subsequent data collection indicating which syringe should be labeled 'inject' or 'discard'. The clinical nurse or assistant confirms the use of the opaque syringe marked 'inject' by the physician performing the injection. The physician, the research staff conducting follow-up interviews, and the patient are therefore all blinded to treatment received.

### Procedure

The procedure is performed in a fluoroscopy suite under strict aseptic conditions. The patient is placed prone on the procedure table. Standard fluoroscopy is used for localization of the spinal/vertebral level to be treated and needle placement for the injection. Fluoroscopic target identification and needle tip entry into the targeted anatomic space is done using previously described procedural techniques [23]. The skin over the back is prepped and draped in a sterile manner using Betadine or chlorhexidine. Local anesthesia with 0.5%-1% lidocaine is injected in the subcutaneous tissues. The epidural and local anesthetic injections are performed using a 20-25 gauge spinal Quinke or 17-20 gauge Tuohy needle. Appropriate needle tip placement is confirmed by fluoroscopy with the injection of 0.5-1.5 cc of iopamadol, ioxehol or equivalent contrast. A fluoroscopic image is obtained following the injection to demonstrate washout of the contrast. This image is saved in the research file. Practitioners are instructed to choose the injection level (e.g., L5-S1) to correspond with one spinal level below the site of maximal canal stenosis for the interlaminar approach injections and at the root level of the greatest symptoms for transforaminal injections. Once an approach (transforaminal versus interlaminar) is chosen for an individual patient, the approach remains consistent for any repeat procedures throughout the trial.

Epidural steroid injectate is 1-3 ml of 0.25-1% lidocaine followed by 1.5-3 ml of 40 mg/ml triamcinolone (i.e. 60-120 mg triamcinolone) in an opaque syringe. The choice of which steroid to use is at the discretion of the treating physician based on his or her usual clinical practice. Betamethasone (6-12 mg), dexamethasone (8-10 mg) or methylprednisolone (60-120 mg) may also be used. The needles are then removed and the patient taken to the recovery area.

The control procedure is identical to the ESI except that the epidural injectate is 1-3 cc of .25%-1% lidocaine followed by 1.5-3 cc of .25%-1% lidocaine in an opaque syringe. The total volume of injectate is the same for the ESI and the control injection to avoid differential treatment effects based on volume.

### Blinding

Every effort is made to maintain blinding of the patients and evaluators during the course of the trial. All baseline and follow up interviews are conducted by a study research assistant who remains blinded to the randomized assignment of the patient throughout the study. The research assistant also completes assessments of blinding at all follow up time points. All datasets have the randomization code masked until all pre-specified analyses are conducted to ensure that the research team remains unaware of patient assignment. Patients are asked to guess which treatment they actually received at 2 weeks, 5 weeks, and 3 months after the initially randomized injection ("ESI", "local anesthetic", or "don't know") and to provide a reason for and confidence in treatment guesses other than "don't know". Specific instances of un-blinding will be recorded and the study statistician will monitor treatment guesses to diagnose systematic problems with the blinding procedure or over the course of conducting the study. Using Fisher's exact test, we will formally compare treatment guesses between arms 2 weeks after randomization when the study results are reported.

### Analytic approach

We are utilizing "intent-to-treat" [24] analyses for the primary effectiveness evaluation, which evaluates outcomes based on the treatment arm to which individuals are assigned. Crossover is not allowed until after the outcome measures have been collected at the study primary endpoint of 6 weeks; therefore, the primary results will reflect the treatment to which the participant was randomized. The evaluable patient subset is defined as those patients with data available for analysis based on their initially randomized treatment. Consideration of missing data is essential to characterize the potential for selection bias through attrition. Characteristics of patients with missing follow-up data will be compared to those with complete data. In sensitivity analyses, we will impute missing outcome data in multiple ways to assess its impact on the primary analyses.

### Primary analyses

Our primary analysis will evaluate the effect of ESI + local anesthetic compared to local anesthetic alone using repeated measures analysis of covariance (ANCOVA). Each ANCOVA model will adjust for the respective outcomes measured at baseline and a dummy variable indicating study recruitment site. From the ANCOVA models, we will report on the means and standard deviations of six-week outcome measures for each group and an adjusted estimate of the mean difference (95% CI) between groups. We will adjust the primary analysis for important baseline characteristics that, despite randomization, differ between groups and are associated with the outcomes.

### Secondary analyses

We will conduct two subgroup analyses to assess evidence for treatment effect on the primary outcome measures within categories defined by race and injection approach (transforaminal/interlaminar). We do not hypothesize that the treatment effect will be larger or smaller in one subgroup compared to another, only that there is a significant treatment effect within each subgroup.

We will assess secondary outcomes using ANCOVA models for continuous measures and logistic regression models for binary measures. These analyses will include assessment of group differences in clinically meaningful changes in pain and function (e.g., at least a 30% improvement on the Roland Morris Disability Questionnaire and pain scores).

Secondary analyses will consider the complete time profile for the primary outcome measures of pain and function through 12 months using methods appropriate for the analysis of repeated measures. Crossover and missing data will complicate analysis of follow-up data. Long-term outcomes will be evaluated using linear mixed effects models for repeated measures in both intent-to-treat and as-treated analyses. We will tabulate crossover rates by randomized treatment arm and compare the rates using Chi-squared tests and logistic regression as appropriate. To assess the impact of crossover on the overall evaluation of ESI, we will examine longitudinal models of outcome that incorporate a time-varying covariate indicating when treatment occurred.

### Economic outcome measures

The economic evaluation will include an assessment of health care utilization, costs, and cost-effectiveness for the steroid group compared to those receiving local anesthetic alone in the intention-to-treat sample. The co-primary economic outcomes will be incremental per participant average costs compared to per participant Roland Morris Disability Questionnaire improvement and incremental cost per quality-adjusted life year (QALY) gained from both a payer and societal perspective [25]. The QALY will be calculated using the EQ-5D, a standardized health outcome instrument used to estimate "utility weights" for cost-utility analysis http://www.euroqol.org. We will estimate incremental cost-effectiveness ratios for six weeks, six months and one year using advanced sensitivity analyses to adjust parameter ranges for uncertainty in clinical, cost, or effectiveness variables [26]. The impact of complications and adverse effects on resource utilization and costs will be summarized for both groups.

The primary economic research question is whether treatment with steroid combined with anesthetic will be safer and more effective than treatment with local anesthetic alone, at a differential cost within willingness-to-pay thresholds of U.S. payers, using an incremental cost per QALY gained approach. The one-year societal perspective analysis will include direct and indirect costs, using standardized payer-based reimbursement amounts (i.e., unit costs). In summarizing overall costs, we will include costs reported by participants via resource use questionnaires, in addition to the sum of estimated reimbursed amounts associated with care used. Within-health-system resource utilization will be assessed using clinical, pharmacy and administrative data from the participating health plans' electronic systems. We will use the health plan data to assess medical utilization events (e.g., office visits, surgeries, tests) and the pharmacy data to assess filled prescriptions. Average unit prices for resource use (pharmacy and medical) will be applied to participants' health system utilization using a reimbursement database containing payer expenditures within each health system. These unit cost estimates (i.e., payer reimbursement amounts) will come from an independent, private insurance claims data warehouse, i.e., Marketscan^® ^[27].

The secondary economic question is whether steroid injections represent good value for money as evidenced by non-QALY incremental cost-effectiveness ratios (i.e., cost per improvement in back-related functioning and cost per improvement in back pain, using Roland Morris Disability Questionnaire and other pain/function measures). Other consequences (outcomes) will be described in relation to cost or use profiles (e.g., ranges of the Roland Morris Disability Questionnaire, pain numeric rating scale measures, categories of medication use, imaging or other diagnostic testing, and other provider use). Marketscan^® ^data will be used for unit cost estimates.

During four, three-week time periods over the course of the twelve month study (weeks: 1-3, 4-6, 24-26, and 50-52), participants will complete Resource Utilization Questionnaires to record information related to out-of-pocket (out-of-system) costs, time related to seeking back pain treatment, daily use of prescription and over-the-counter medication use, and additional back therapies, along with use or purchases of other back-care products. We will describe the participant-reported data by randomized groups. Additional descriptive analyses will include assessing treatment group differences in dis-aggregated EQ-5D items representing activities of daily living (mobility, self-care, usual activities, pain/discomfort, anxiety/depression).

The effectiveness measures for the cost-effectiveness analysis will be obtained directly from the primary statistical analysis. The cost-effectiveness assessment will use a decision-tree framework to quantitatively assess the costs and effects for the randomized groups. Short-term cost-effectiveness outcomes will use the intention-to-treat approach, while longer-term, one-year outcomes will use empirical approaches to estimate EQ-5D and Roland Morris Disability Questionnaire outcomes in relation to cost. The assessment will allow for different resource use patterns and test ranges of assumptions for treatments and procedures. Empirical data from LESS and from external sources which included the relevant outcome measures [28,29] will be used to estimate the expected effects and costs for ESI-randomized participants, modeling outcomes of Roland Morris Disability Questionnaire and EQ-5D at three, six, and 12 months. These data will be used to model treatment groups under the assumption that no crossover occurs at the six-week endpoint and participants do not deviate from their randomized treatment during the 12-month study, rather than modeling diverse crossover treatment patterns and associated outcomes for participants, which will be presented descriptively. Data on improvement or decrement in outcomes will be used to estimate the trajectory of Roland Morris Disability Questionnaire or EQ-5D for treatment groups. Presenting ranges of cost-effectiveness estimates using different approaches will allow for a "triangulation" of results to assess consistency, so that payers and policy makers can have comprehensive information for decision-making. The analysis will be influenced by published data on treatment patterns and guidelines, in addition to assessing trends in Marketscan^® ^data during several years. Costs will be estimated in 2013 US$, the year the study will conclude. As is standard practice in cost-effectiveness analyses of 12 months or less, no discounting of costs or effects will be performed for the 12-month study. Sensitivity analyses will be conducted by testing a 20% range versus base case values for cost parameters and effectiveness measures, as well as using probabilistic sensitivity analysis approaches [26]. Additional testing will be performed on cost-effectiveness parameters having the greatest impact on the variability of cost-effectiveness estimates.

### Sample size

Our target is to enroll up to 400 patients with half randomized to ESI + local anesthetic and half to local anesthetic alone. We determined overall sample size based on the primary outcome (Roland Morris Disability Questionnaire) with two subgroup analyses (race: Caucasian/non-Caucasian; approach: interlaminar/transforaminal). We will use ANCOVA as the primary method of analysis with the following parameters: 85% follow-up at 6 weeks; two-sided Type 1 error rate of α = 0.05; and adjustment for recruitment site and the outcome measured at baseline. For the purpose of sample size estimation, we conservatively assume six-week Roland Morris Disability Questionnaire scores will have a standard deviation of 7.5 for each group and a correlation between baseline scores and six-week scores of 0.3. For the combined (n = 400) assessment of ESI + local anesthetic vs. local anesthetic alone, the LESS study has 83% power to detect between-group mean differences as small as 2.25 points on the Roland Morris Disability Questionnaire.

Subgroup analyses were powered to detect clinically meaning differences between treatments *within *each subgroup; we did not power the study for the hypothesis of a larger treatment effect within one subgroup compared to another (e.g. an interaction). We adjusted the overall two-sided Type 1 error rate of α = 0.05 using a Bonferroni correction for two tests of the data. Using ANCOVA methods, a sample size of n = 200 (100 per group) provides 83% power to detect between-group mean differences of 3.25 points on Roland Morris Disability Questionnaire.

While formal interim analyses of treatment effect are not planned for this study, we will generate blinded variance estimates for the primary outcomes measured at 6 weeks after a minimum of 100 patients have been enrolled. Conservative estimates of outcome variance and 6-week retention utilized in the initial design of the study yielded an overall upper-bound sample size of n = 400. Adaptive sample size re-estimation will be conducted to provide more precise overall sample size requirements given the observed variance and retention rate, while maintaining the overall Type 1 error rate and power for the stated effect sizes [30].

## Discussion

As there has been scant research focusing on ESI in older adults with pain due to spinal stenosis, little is known about treatment risks and benefits for this population. Although it has been demonstrated that surgery can be effective for severe spinal stenosis, there are many elderly patients for whom surgery carries substantial risk. There is a need for safer, less-invasive treatment options for older adults with the hope of reducing functional disability and promoting independence. Although ESI is a promising treatment alternative and is widely used in clinical practice, its effectiveness is unknown. Compared to young and middle-age adults, a much higher percentage of older adults have co-morbid conditions such as diabetes that may increase the risks due to systemic absorption of corticosteroids.

As in most clinical trials, we anticipate that recruitment may be a challenge. Patients suffering from pain may be reluctant to delay what they might consider to be definitive therapy. We have developed strategies to increase our chances of successful recruitment including the use of a patient-education video, which was a successful strategy in our prior trials [29]. We have multiple clinical recruitment sites and have added additional clinical sites to attain our recruitment targets. We also have included an optional crossover design to maximize our ability to recruit patients with severe pain into the trial.

The results of this study should provide compelling evidence as to whether or not epidural steroid injections are effective in improving function and pain in the elderly with lumbar spinal stenosis. The LESS trial will provide high-quality data for shorter-term outcomes (six weeks) and longer-term (one-year) outcomes. The patient-reported outcomes, the electronic medical and pharmacy utilization records, and supporting economic data providing reimbursed amounts for resources used will enable a robust evaluation of the comparative benefits and harms associated with epidural steroid injections with anesthetic versus local anesthetic alone.

## Abbreviations

ANCOVA: Analysis of co-variance; BOLD: Back pain Outcomes using Longitudinal Data; BPI: Brief Pain Inventory Interference; CEA: Cost effectiveness analysis; ESI: Epidural steroid injection; GAD-7: Generalized Anxiety Disorder measure; LESS: Lumbar Epidural steroid injections for Spinal Stenosis; PHQ-8: Patient Health Questionnaire (PHQ-8); SSSQ: Swiss Spinal Stenosis Questionnaire (SSSQ); NRS: Numerical rating scale; QALY: Quality adjusted life year.

## Competing interests

The authors declare that they have no competing interests.

## Authors' contributions

All authors contributed to development of the research protocol, manuscript writing and editing. All authors read and approved the final manuscript.

## Pre-publication history

The pre-publication history for this paper can be accessed here:

http://www.biomedcentral.com/1471-2474/13/48/prepub
